# The Effect of Ranibizumab on Normal Neurosensory Retina in the Eyes of Patients with Exudative Age Related Macular Degeneration

**DOI:** 10.2174/1874364101711010368

**Published:** 2017-12-29

**Authors:** Olga E. Makri, Demetrios Vavvas, Panagiotis Plotas, Athina Pallikari, Constantine D. Georgakopoulos

**Affiliations:** 1 Department of Ophthalmology, Medical School, University of Patras, Patra, Greece; 2 Massachusetts Eye and Ear Infirmary, Department of Ophthalmology, Harvard Medical School, Boston, MA, USA

**Keywords:** Age related macular degeneration, Ganglion cells, Neurosensory retina thickness, Ranibizumab, Toxicity, AMD

## Abstract

**Background:**

Anti-vascular endothelial growth factors have become the mainstay treatment for neovascular age related macular degeneration. Prolonged suppression of vascular endothelial growth factor raises concerns as it may result in harmful effects on retina.

**Objective:**

The purpose of this retrospective chart review is to evaluate the 1-year effect of treatment with intravitreal injections of ranibizumab on normal neurosensory retinal tissue of patients with exudative age related macular degeneration using the Optical Coherence Tomography (OCT).

**Method:**

The study included **s**ixty five eyes of 62 patients (32 male and 30 female; mean age 74.97±8.5 years) with exudative age related macular degeneration treated with intravitreal injections of ranibizumab with a *pro re nata* treatment regimen over a period of 1 year. The MM5 thickness maps acquired with the Optovue RTVue-100 Fourier-domain OCT at baseline, at 3 months, after the 3 loading doses of ranibizumab, and at the 1 year follow-up visit were used for analysis. Changes of inner and outer retinal thickness in four selected points of normal retina on the MM5 scan were evaluated.

**Results:**

The patients received a mean of 6.4 ± 1.8 (median 6, range 3-11) intravitreal injections of ranibizumab over a period of 12 months. No significant change was observed in inner and outer retinal thickness at pre-selected spots of normal retina during the first year of intravitreal administration of ranibizumab.

**Conclusion:**

One year treatment with ranibizumab on an individualized, according to need dosing regimen does not seem to induce any detectable structural damage in the unaffected, normal retina.

## 1 INTRODUCTION

Age related macular degeneration (AMD) is the leading cause of loss of central vision among elderly people worldwide [1]. The introduction of pharmaceuticals that inhibit Vascular Endothelial Growth Factor (VEGF) has revolutionized the management and has become the mainstay treatment for exudative or neovascular AMD (nvAMD) [2]. Ranibizumab is a high-affinity humanized recombinant monoclonal antibody fragment that neutralizes all VEGF-A isoforms. It has been approved as a treatment for nvAMD, diabetic macular edema, macular edema secondary to retinal vein occlusion and visual impairment due to myopic choroidal neovascularization (CNV) or other types of CNV [3].

Management of nvAMD requires prolonged periods of treatment with multiple intravitreal injections of anti-VEGF agents. However, results from experimental studies have highlighted the neurotrophic, neuroprotective and neuroregenerative properties of VEGF-A in a variety of nervous tissues and cells, including photoreceptors and ganglion cells, indicating the critical role of VEGF-A in the adult retina [4-6]. Therefore, prolonged suppression of VEGF with anti-VEGFs like ranibizumab has become an important consideration as it may result in harmful downstream effects on retinal tissue.

After 2 years of monthly ranibizumab administration for nvAMD, 9% and 10% of participated patients in the phase III MARINA and ANCHOR studies, respectively, had lost at least 15 letters accompanied by an increase in retinal pigment epithelium (RPE) pigmentary disturbances and with increase in the area of the lesion. The authors attributed this observation to photoreceptor loss from the process of the CNV itself while they could not exclude the plausibility of ranibizumab toxicity due to the suppression of VEGF and its neuroprotective role [7]. Horsley *et al*. found no significant change on the mean peripapillary retinal nerve fiber layer (RNFL) thickness in patients with nvAMD treated with an average of 13.4 intravitreal injections of ranibizumab over a period of about 20 months [8]. Similarly, total and nasal RNFL thickness was not adversely affected in patients that had been administered at least 10 ranibizumab injections [9]. However, different results were found from Martinez-de-la-Casa *et al*. who in a prospective controlled study found that treatment with a mean of 4.8 intravitreal injections of ranibizumab over a period of 12 months resulted in significant RNFL thinning [10]. The RNFL evaluation is a surrogate marker of axonal health of retinal ganglion cells (RGCs) in clinical field and RNFL thinning in patients administered ranibizumab could be attributed to the Intraocular Pressure (IOP) fluctuations or to the neurotoxic effect of ranibizumab itself on ganglion cells [11].

Consequently, there is an increasing concern about the possible toxic effects of prolonged inhibition of VEGF on healthy intraocular tissues of eyes undergoing repeated intravitreal injections of ranibizumab. The purpose of our study was to investigate ranibizumab's possible toxicity in neurosensory retina in patients with nvAMD treated with ranibizumab on an as needed [*pro re nata* (PRN)] basis over a period of 12 months. The thickness of normal areas of neurosensory retina was used as a potential objective morphologic sign of retinal toxicity.

## 2 MATERIAL AND METHODS

This is a single-center retrospective chart review of patients treated with intravitreal injections of ranibizumab for nvAMD in the medical retina division of the Department of Ophthalmology of the University Hospital of Patras. The Institutional Review Board of the University Hospital of Patras approved the study with a waiver of informed consent.

Eligible patients were treatment naive patients diagnosed with nvAMD and treated for at least 1 year with intravitreal injections of 0.05 mL of 10 mg/mL solution of ranibizumab (Lucentis; Novartis Pharma S.A.S., Huningue, France) on a PRN basis. According to that treatment scheme, 3 monthly injections are administered as a loading dose and thereafter retreatment is given if best-corrected visual acuity (BCVA) is decreased compared with the previous visit or if signs of active CNV are found, like new hemorrhage on dilated fundus examination or fluid accumulation on Optical Coherence Tomography (OCT) examination [12]. Eligible patients should also have been examined with the MM5 grid scanning protocol of RTVue-100 Fourier-domain OCT (software version 5.1.0.90; Optovue Inc., Fremont, CA, USA) before ranibizumab administration and at 3 months, after the 3 loading doses of ranibizumab, and at the 12-month visit.

Exclusion criteria were any pervious treatment with other anti-VEGF agents like pegaptanib, bevacizumab or aflibercept and history of photodynamic therapy or thermal laser photocoagulation for CNV. Patients with anomalies of the vitreoretinal interface and other phenotypes of nvAMD, like retinal angiomatous proliferation, polypoidal choroidal vasculopathy were not eligible for the study. Cases with lesions that extended to the boundaries of the MM5 measurements were also excluded. Furthermore, patients with diabetes, pseudoexfoliation syndrome, ocular hypertension, glaucoma or other optic nerve disease or any ocular inflammation were excluded. Any history of vitreoretinal surgery at any timepoint or history of cataract surgery within 6 months of treatment initiation were also exclusion criteria.

Medical records of the eligible patients were reviewed for age, sex, treated eye, IOP at baseline and last examination and total number of administered intravitreal injections of ranibizumab. Baseline BCVA and BCVA at the 3 and 12 month examination were reviewed and converted from Snellen fraction to a logarithm of the minimum angle of resolution (logMAR) for statistical analysis. The MM5 measurements of the OCT at baseline and at the 3 and 12 month visit were used for analysis. The MM5 grid scanning protocol consists of 11 horizontal and 11 vertical lines with 668 A-scans each that cover a 5x5 mm^2^ square area and an inner 3x3 mm^2^ square region of 6 horizontal and 6 vertical lines with 400 A-scans each [13]. Using the analysis program of the MM5 protocol we can generate retinal thickness data of each selected point of the scanned area. The segmentation algorithm of the MM5 scanning protocol also enables the automatic segmentation of the total retinal thickness to Inner Retinal Thickness (IRT) and Outer Retinal Thickness (ORT). According to the instrument’s user's manual the ORT is measured from the IS/OS layer to the inner plexiform layer (IPL), while IRT represents the thickness from vitreoretinal interface to the IPL [13].

Each scan was carefully reviewed for the accurate identification and segmentation of the retinal layers. All scans were evaluated to ensure that baseline and follow-up images were centered on the fovea and the en face imaging of retinal blood vessels and of the optic nerve head in the SLO image was used as landmark in order to confirm that the baseline and follow up MM5 scans refer to, as much as possible, the exact same location of the posterior pole [14]. The progression analysis report was also used to ensure that the scanned areas coincided in the baseline and follow up examinations. The OCT image quality signal strength index of the acquired scan should be at least 40. Patients with MM5 scans that did not fulfill the above criteria were excluded from the study.

Four points were selected from the MM5 grid scan; one superior temporal (ST), one inferior temporal (IT) point, one Inferior Nasal (IN) and one Superior Nasal (SN) spot Fig. (**1**). Each one of these points was located at a precise distance from the fovea and more specifically at the four corners of a 4x4 mm^2^ square region of the MM5 scan. Scrolling the tip of the cursor at each point, it is defined with the following coordinates; [-2.00, 2.00] mm the ST, [-2.00, -2.00] mm the IT, [2.00, -2.00] mm the IN and [2.00, 2.00] mm the SN point while the fovea is determined as [0.00, 0.00] mm. The selected points should refer to normal retina at baseline and during the 12 month period and, ideally, all the four points were far from the pathology of the nvAMD. Any point that was located within a region with pathology was excluded from the analysis. Clicking on each spot the corresponding IRT and ORT values were measured at the baseline, at the 3-month visit, after the 3 loading doses of ranibizumab, and at the 12-month MM5 scan. Any change of the IRT and ORT for each point was evaluated.

## 3 STATISTICAL ANALYSIS

Statistical analysis was performed with SPSS 21.0 software (SPSS, Inc., Chicago, IL). The sample size in this study was calculated as at least 30 patients with a significance level ≤ 0.05, and a statistical power of 0.9. The sample size calculation was based on the results of similar prior study [10]. All variables were tested for normality with the Kolmogorov-Smirnov test. The paired sample Student's *t*-test was performed for pair-wise comparisons of parametric values. Repeated measures ANOVA was used to compare parametric values with Bonferroni post hoc test for comparisons between values within subjects. Friedman non-parametric test was used to compare repeated measures for non parametric values. Wilcoxon signed-rank tests were conducted for post hoc comparisons between observations within subjects. A level of p < 0.05 was considered to be statistically significant.

## 4 RESULTS

Records were available for 157 patients who had received intravitreal injections of anti-VEGF agents for nvAMD between March 2009 and January 2016. Sixty five eyes (31 right and 34 left eyes) of 65 patients (31 male and 34 female; mean age 74.97 ± 8.5 years) fulfilled all the inclusion/exclusion criteria and were included in the study. The patients received a mean number of 6.4 ± 1.8 (median 6, range 3-11) intravitreal injections of ranibizumab over a period of 12 months. Mean BCVA at 12 months visit was statistically significantly higher compared to baseline (p = 0.036). The mean IOP at the 12-month examination did not differ significantly from the baseline IOP (p = 0.155). No ocular or systemic adverse events were reported during the treatment period. Characteristics of the patients included in our study are summarized in (Table **1**).

The average IRT and ORT measured at baseline, after the three loading doses of ranibizumab and at the 12 month examination in the 4 selected extrafoveal points of normal retina are provided in Table (**2** and **3**). Although there was a minimal trend of reduced IRT and ORT, no statistical significant change was observed in IRT or ORT at the selected spots of normal retina during the first year of intravitreal administration of ranibizumab (Table **2** and **3**).

## 5 DISCUSSION

During the last decade, treatment with intravitreal injections of anti-VEGF agents like ranibizumab has become the standard of care for nvAMD [15]. Their administration on a PRN basis seems to have excellent results with significant improvement of VA [12]. Ranibizumab seems to have a favorable safety profile with rare ocular and systemic adverse events [16, 17].

Treatment with ranibizumab, however, results in VEGF inhibition, an agent essential for several vital functions. During embryogenesis VEGF acts as endothelial cell growth factor necessary for the development of normal blood vessels [18], while it has also been shown to have neurotrophic, neuroprotective and neuroregenerative properties in the central nervous system and in the eyes of adult animals [4-6]. Vascular endothelial growth factor has been detected in ganglion cell layer (GCL), inner nuclear layer (INL), the RPE as well as in a subset of cone photoreceptors. Using a model of ischemia-reperfusion injury model in rats, Nishijima *et al.* showed that VEGF receptor-2 (VEGFR-2), the primary signaling receptor of VEGF is highly expressed as adaptive response to ischemic injury, in the retinal vascular endothelial cells and in the neuronal cells in both the GCL and the INL [19]. Another study showed that VEGFR-2 is strongly expressed in Müller cells and photoreceptors of the normal adult mouse retina [6]. The expression of VEGF and VEGFR-2 in animal's normal adult eyes denotes a potential endogenous protective, functional or survival role for VEGF-A in the normal retina.

Several experimental studies tried to evaluate the effect of VEGF-A blockade and resulted in controversial conclusions. Targeted inactivation of VEGF-A in the RPE of adult mice resulted in extensive atrophy of the choriocapillaris, marked attenuation of choroid vessels and dysfunction of cone photoreceptors [20]. The neuroprotective role of VEGF-A on RGCs was demonstrated in an experimental animal model of glaucoma that showed that VEGF-A acts directly on RGCs promoting cell survival and that antagonism of VEGF-A function significantly increased the rate of RGCs apoptosis [21]. Intravitreal treatment with VEGF demonstrated protective effect and reduced the apoptosis of RGCs in rats with chronic intraocular hypertension [22]. On the other hand, another *in vivo* study showed that intravitreally administered anti-VEGFs like bavacizumab and ranibizumab, even at higher doses, had no toxic effect on RGCs of normal rat eyes [23]. Miki *et al.* have also demonstrated that prolonged blockade of VEGF receptors had no toxic effect on normal retina, as this was evaluated with examination of the outer nuclear layer (ONL), RGCs and photoreceptors [24]. Nevertheless, another study showed a direct survival function for VEGF on Müller cells and photoreceptors and also demonstrated that systemic neutralization of VEGF in mice resulted in increased apoptosis in the INL and ONL associated with retinal function impairment [6].

In light of data from these animal models concerns have been raised for possible risk of chronic anti-VEGF administration in human eyes. This study retrospectively evaluated the effect of intravitreal injections of ranibizumab on normal retinal tissue in patients with nvAMD treated with ranibizumab for 12 months on a PRN basis. The ORT and IRT at precise points of normal neurosensory retina were used as an objective parameter, using the Fourier domain OCT (RTVue-100) with the MM5 scanning protocol which has demonstrated high reproducibility [25].

The measurement of ORT corresponds to the thickness from the ellipsoid zone to the IPL that includes, among others, the cell bodies of the photoreceptors and the horizontal, bipolar and amacrine cells of the retina. The IRT of the MM5 protocol represents the thickness measured from vitreoretinal interface to IPL and includes the nuclei of the ganglion cells and the layer of optic nerve fibers. According to our results none of the above structures were affected, as far as morphology/thickness is concerned, in 4 pre-selected points of normal retina after 12-month treatment with intravitreal injections of ranibizumab in a PRN dosing regimen.

To date, several clinical studies, with controversial results, tried to evaluate the safety of intravitreal ranibizumab injections in human eyes and to focus on the cumulative long-term risk of repeated ranibizumab injections in regard to normal human retina. A recent meta-analysis, that included 288 eyes with nvAMD treated with anti-VEGF injections administered on a PRN basis on non-glaucomatic patients, found no association between anti-VEGF injections and RNFL thickness changes [11]. However, when two low-biased, nonrandomized, controlled prospective clinical trials including 71 eyes were examined separately it was found a significant association between repeated ranibizumab injections and the risk for RNFL thinning [11]. A retrospective study by Saleh *et al.* that was not included in the abovementioned meta-analysis tried to evaluate the effects of administration of 39 ± 16 anti-VEGF agents over a period of 6.1 ± 2.1 years on the RNFL and GCL thickness in 16 glaucomatic patients with nvAMD [26]. The authors found no significant changes on mean RNFL thickness while the GCL thickness decreased significantly in correlation to the number of the administered intravitreal injections [26]. Focused on RNFL and GCL thickness Beck *et al.* conducted a retrospective case series study with fellow-eye comparison in 38 patients with nvAMD administered a mean number of 31.5 anti-VEGF injections over a mean period of 45.3 months and found that the RGCL thickness decreased significantly compared to the noninjected fellow eye, while the decrease of the RNFL was not significant [27]. The safety of ranibizumab was examined electrophysiologically in 32 patients with nvAMD who underwent a mean of 4.09 of intravitreal injections of ranibizumab over a period of 12 months. The results from full field photopic electroretinogram showed that the function of the retinal neurons in the cone pathway, that is cone photoreceptors, cone bipolar cells, amacrine cells, and retinal ganglion cells of the entire retina, was not affected by repeated ranibizumab administration [28].

Our objective was to evaluate any possible toxic effect of intravitreal injections of ranibizumab on healthy retinal tissue. Precise points at the posterior pole, representative of normal retinal tissue, were selected and the thickness of their layers was used as indicator in order to evaluate any retinal toxicity of ranibizumab. Using the quantitative assessment of retinal thickness via the MM5 scan of the RTVue OCT we found that during the first year of PRN treatment for nvAMD with ranibizumab the retinal thickness of specific spots of normal retina was not affected significantly. It should also be pointed out that intravitreal injections of ranibizumab did not result in significant change in the IOP during the period of study, whereas transient IOP spikes after the intravitreal injection could not be excluded.

However, we have to admit that our study has certain methodological limitations like the retrospective study design, the relative small sample size and the lack of control group. Follow-up time of 12 months is certainly a short period to evaluate possible toxicity of ranibizumab on neurosensory retina and a study of a longer treatment period could have provided more solid conclusions. Furthermore, we could compare changes of the ORT and IRT in injected eyes to those in the untreated fellow eyes. We also recognize that it may be fairly difficult to obtain images of adequate quality in patients who present with poor VA. However, the majority of the patients included in the study had unilateral involvement with the other eye retaining satisfactory central vision allowing appropriate fixation and centration of the MM5 scan to the fovea, even with the use of the external fixation light. Moreover, we focused on isolated points in the retina and we used and compared examinations conducted at different visits. The OCT used lacks the implementation of an eye-tracking system that enables accurate and repeatable alignment of OCT images, a feature that would provide even more accurate results. Finally, utilizing the available technology of the specific SD OCT equipment we were not able to apply precise segmentation of retinal layers and to evaluate possible changes at each separate layer of the retina while we could not assess at all any morphological changes in the RPE.

## CONCLUSION

There still a lot of unanswered questions regarding the possible side-effects of long term inhibition of the VEGF in the human eye. Several experimental models have highlighted the neurotrophic effect of VEGF in the animal retina and pointed out the possible deleterious effects of chronic intravitreal treatments with anti-VEGF agents. Nevertheless, only a few studies have focused on the possible toxic effect of anti-VEGF agents on the normal human retina with conflicting data. We have examined patients with nvAMD and found that 1-year therapy with intravitreal injections of ranibizumab on a PRN basis does not appear to affect morphologically the normal human neurosensory retina. Well-designed, prospective studies recruiting larger number of patients with longer follow-up time and probably utilizing OCT with precise segmentation are required to confirm our findings in order to draw safe conclusions regarding the long-term safety of anti-VEGF treatments for the normal retina.

## Figures and Tables

**Fig. (1):**
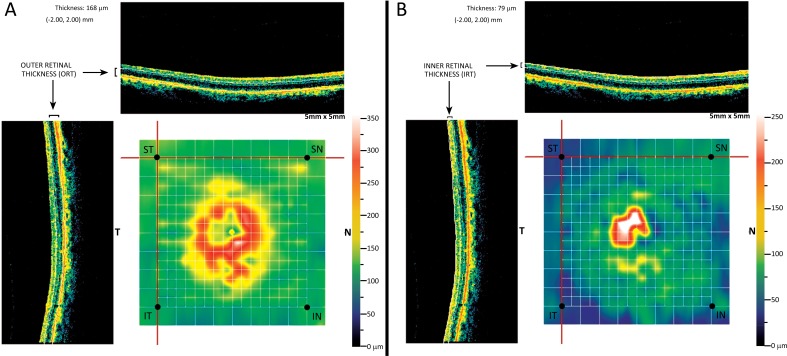
A representative MM5 grid scan of a patient that was used for analysis. One superior temporal (ST), one inferior temporal, one inferior nasal and one superior nasal spot were selected in the MM5 grid scan. The segmentation algorithm of the MM5 scanning protocol enables the automatic segmentation of the total retinal thickness to outer retinal thickness (ORT) and inner retinal thickness (IRT). In the depicted scans of the patient the ORT (A) and the IRT (B) were evaluated in the selected ST spot, which is defined with the [-2.00,2.00] mm coordinates . The ORT is 168μm while the IRT is measured 79μm in the ST spot of the particular patient. The selected points refer to normal retina.

**Table 1:** Characteristics of the patients included in the study.

Patients Characteristic	
Number of patients	65
Age at baseline (mean ± SD), years	74.97 ± 8.5
Sex (Male, Female)	31, 34
Study eye (Right, Left)	31, 34
Number of ranibizumab injections (mean ± SD)	6.4 ± 1.8
Best-corrected visual acuity (logMAR) [median (range)]	
Baseline	0.70 (0.1-3.0)
3 months	0.40 (0-3.0)
12 months	0.30 (0-3.0)*
Intraocular pressure (mean ± SD)	
Baseline	15.72 ± 3.65
12 months	16.22 ± 3.03

*Statistically significantly higher compared to baseline (p = 0.036)

**Table 2:** Inner retinal thickness measurements. The average inner retinal thickness measured at baseline, after the three loading doses of ranibizumab and at the 12 month examination in the 4 selected extrafoveal points of normal retina. Parentheses denote the number of patients that were evaluated at each point.

**Inner retinal thickness**				
	**Baseline**	**Three Months**	**Twelve Months**	**p**
**Superior temporal** (57)	84.09 ± 11.91	81.41 ± 12.39	81.00 ± 13.18	.147
**Inferior temporal** (60)	84.77 ± 12.57	81.97 ± 13.60	83.13 ± 14.25	.255
**Superior nasal** (55)	101.93 ± 13.22	101.70 ± 10.48	99.20 ± 13.99	.450
**Inferior nasal** (62)	101.00 ± 12.90	100.30 ± 13.68	96.63 ± 17.54	.099

**Table 3:** Outer retinal thickness measurements. The average outer retinal thickness measured at baseline, after the three loading doses of ranibizumab and at the 12 month examination in the 4 selected extrafoveal points of normal retina. Parentheses denote the number of patients that were evaluated at each point.

**Outer retinal thickness**				
	**Baseline**	**Three Months**	**Twelve Months**	**p**
**Superior temporal** (57)	157.82 ± 11.75	155.38 ± 12.14	157.56 ± 12.93	.375
**Inferior temporal** (60)	154.50 ± 7.57	153.20 ± 10.93	153.83 ± 10.25	.741
**Superior nasal** (55)	165.97 ± 15.27	163.47 ± 13.30	163.23 ± 11.62	.372
**Inferior nasal** (62)	164.47 ± 15.59	162.77 ± 14.05	160.43 ± 13.67	.065

## LIST OF ABBREVIATIONS

AMD: = Age related macular degeneration
BCVA: = Best corrected visual acuity
CNV: = Choroidal neovascularization
GCL: = Ganglion cell layer
IN: = Inferior nasal
INL: = Inner nuclear layer
IOP: = Intraocular pressure
IPL: = Inner plexiform layer
IRT: = Inner retinal thickness
IT: = Inferior temporal
logMAR: = Logarithm of the minimum angle of resolution
nvAMD: = Neovascular AMD
OCT: = Optical coherence tomography
ONL: = Outer nuclear layer
ORT: = Outer retinal thickness
PRN: = *pro re nata*
RGCs: = Retinal ganglion cells
RNFL: = Retinal nerve fiber layer
RPE: = Retinal pigment epithelium
SN: = Superior nasal
ST: = Superior temporal
VEGF: = Vascular endothelial growth factor
VEGFR-2: = VEGF receptor-2
